# Chromosomal microarray analysis for pregnancies with abnormal maternal serum screening who undergo invasive prenatal testing

**DOI:** 10.1111/jcmm.16589

**Published:** 2021-05-27

**Authors:** Xiaoqing Wu, Ying Li, Na Lin, Xiaorui Xie, Linjuan Su, Meiying Cai, Yuan Lin, Linshuo Wang, Meiying Wang, Liangpu Xu, Hailong Huang

**Affiliations:** ^1^ Fujian Provincial Key Laboratory for Prenatal Diagnosis and Birth Defect Prenatal Diagnosis Center of Fujian Provincial Maternity and Children Hospital Affiliated Hospital of Fujian Medical University Fuzhou City China

**Keywords:** abnormal maternal serum screening, chromosomal microarray analysis, traditional karyotyping, ultrasound anomalies

## Abstract

Recently, chromosomal microarray analysis (CMA) has been implemented as a first‐tier test in pregnancies with ultrasound anomalies. However, its application for pregnancies with abnormal maternal serum screening (AMSS) only is not widespread. This study evaluated the value of CMA compared to traditional karyotyping in pregnancies with increased risk following first‐ or second‐trimester maternal serum screening. Data from 3973 pregnancies with referral for invasive prenatal testing following AMSS were obtained from April 2016 to May 2020. Routine karyotyping was performed and single nucleotide polymorphism array was recommended. The foetuses were categorized according to the indications as AMSS only (group A) and AMSS with ultrasound anomalies (group B). CMA was performed on 713 prenatal samples. The proportion of women opting for CMA testing in both groups increased over the years. The incremental yield of clinically significant findings for pregnancies with high risk of screening results was similar to that for the foetuses with ultrasound soft markers (*P* > 0.05), but significantly lower than that for the foetuses with structural anomalies (*P <* 0.05). The total frequencies of variants of unknown significance in groups A and B showed no significant difference (*P* > 0.05). CMA should be performed for pregnant women undergoing prenatal invasive testing due to AMSS, especially with high‐risk results, regardless of ultrasound findings.

## 1 INTRODUCTION

Traditional prenatal maternal serum screening has been established for two decades,^1^ contributing to a decrease in the prevalence of Down syndrome and Edwards syndrome. In recent years, non‐invasive prenatal testing (NIPT) has improved screening performance because of its higher sensitivity and specificity than traditional screening tests. Nevertheless, NIPT is costly and not publicly funded, while traditional screening is publicly funded and included in population‐based programme in China. Hence, in the vast majority of countries and territories, the serum screening test remains the predominant method, although the rate of serum screening has decreased after NIPT introduction. Regarding abnormal maternal serum screening (AMSS), further testing recommendations include invasive procedure such as amniocentesis, chorionic villus sampling or a secondary screening tool of high sensitivity like NIPT. Although women with AMSS results are optimal candidates for NIPT,^2^ in practice, considering that confirmatory invasive testing is mandatory for cases with NIPT‐positive results in the end, quite a few women opt for invasive diagnostic testing to obtain definite results.

It has been generally accepted that chromosomal microarray analysis (CMA) should be recommended in pregnancies with foetal structural abnormalities.^3^, ^4^ However, its use for AMSS pregnancies is not yet widespread. Maternal serum screening is mainly targeted to detect chromosomal aneuploidy, including trisomy 21, 18 and 13, and not to detect an increased risk of microdeletions/microduplication syndromes. Due to the absence of a consensus concerning the optimal testing method, pregnant women often hesitate to choose traditional karyotyping alone or to choose CMA testing concurrently undergoing invasive prenatal diagnosis. Several reports have emphasized the importance of CMA testing in populations with a low risk of microdeletions/microduplication syndromes.^5^, ^6^, ^7^ To provide more data, we reviewed the CMA selection trends and testing results in pregnancies with AMSS combined with or without ultrasound findings in our centre for over 5 years.

## 2 MATERIALS AND METHODS

### 2.1 Patients and samples

This retrospective study reviewed 4070 consecutive singleton pregnancies referred for invasive prenatal diagnosis due to abnormal results on first‐ or second‐trimester maternal serum screening, accompanied with or without other clinical indications. AMSS was defined as high risk for Down syndrome (>1/270), critical risk for Down syndrome (1/1000‐1/270), high risk for Edwards syndrome (>1/350) and critical risk for Edwards syndrome (1/1000‐1/350). Individuals who were chromosomal abnormality carriers and had undergone NIPT, as well as those with adverse reproductive history, were excluded. As a result, 3973 cases were enrolled, and the women were classified into two groups following additional indications: AMSS only (group A) and AMSS with ultrasound anomalies (group B). Amniotic fluid was sampled from 3852 (97.0%) of these women during the 18th and 24th gestational weeks; umbilical cord blood was sampled for 94 (2.3%) women during the 25th and 32nd gestational weeks; and chorionic villi samples were collected from the remaining 27 (0.7%) women during the 10th and 14th gestational weeks. The demographic characteristics are presented in Table 1.

**TABLE 1:** Demographic characteristics of the two groups

	Group A (N = 3600)	Group B (N = 373)
Maternal age (y): mean ± SD	29.6 ± 4.0	29.3 ± 4.1
Gestation age at invasive testing (wk): mean ± SD	20.0 ± 2.0	21.8 ± 4.1
Specimen type		
CV (n)	17, 0.5%	10, 2.7%
AF (n)	3534, 98.2%	308, 82.6
UCB (n)	49, 1.4%	55, 14.7%
CMA (n, %)	476, 13.2%	237, 63.5%

Abbreviations: AF, amniotic fluid; CMA, chromosomal microarray analysis; CV, chorionic villi; SD, standard deviation; UCB, umbilical cord blood.

Conventional karyotyping was routinely performed in all cases. CMA was also recommended to all, but it was performed according to the patients' willingness. Pregnancy outcome follow‐up was conducted via clinical records and telephone communication. This study was approved by the local Ethics Committee of the Fujian Maternal and Child Health Hospital. Written informed consent to participate in the study was obtained from each patient.

### 2.2 DNA extraction and CMA platforms

Genomic DNA was extracted from uncultured amniotic fluid, foetal cord blood and CVS using a QIAGEN kit (Qiagen) according to the manufacturer's instructions. Single nucleotide polymorphism array (SNP array) was performed using Affymetrix CytoScan 750K array (Affymetrix Inc.), which includes 200 000 probes for single nucleotide polymorphisms and 550 000 probes for copy number variations (CNVs) distributed across the entire human genome.

### 2.3 CMA data interpretation

To analyse the results, Chromosome Analysis Suite software (Affymetrix) and human genome version GRCh37 (hg19) were used. A resolution was generally applied: gains or losses of ≥400 kb and loss of heterozygosity (ROH) ≥10 Mb. Uniparental disomy (UPD) was reported based on the identification of the region of homozygosity (ROH) covering the entire chromosome. UPDtool was used for genome‐wide detection of UPD within the child‐parent trios to confirm maternal or paternal UPD origin. All detected CNVs were compared with in‐house and national public CNV databases as follows: Database of Genomic Variants (DGV), Database of Chromosome Imbalance and Phenotype in Humans Using Ensemble Resources (DECIPHER), International Standards for Cytogenomic Arrays Consortium and Online Mendelian Inheritance in Man (OMIM).

Balanced structural chromosome aberrations and chromosome polymorphism were not considered. Incremental yield of CMA was defined as the yield of CMA over traditional karyotyping. The CMA results were classified into pathogenic, benign, likely pathogenic, likely benign and variants of unknown significance (VOUS), based on the American College of Medical Genetics (ACMG) definitions,^8^ Idit Maya, MD,^9^ Jill A. Rosenfeld, MS1^10^ and our inner database. Susceptibility loci (SL) for disease with penetrance over 10% (SL > 10%), foetuses of X‐linked ichthyosis (XLI) in female were categorized as likely pathogenic. As for ROH involving chromosomes that do not involve known imprinted genes, we generally define them as incidental findings. All of these results have been reported for patients. Clinically significant aberrations refer to pathogenic and likely pathogenic results. Parental CMA was recommended to determine the inheritance of CNVs. In general, CNVs inherited from normal phenotype parents were regarded as likely benign.

### 2.4 Conventional karyotyping

This procedure was described in our previous study.^11^

### 2.5 Statistical analysis

The data were analysed using SPSS software v19.0 (SPSS Inc.). Statistical comparisons were performed using the chi‐square test, and *P* < 0.05 was considered statistically significant.

## 3 RESULTS

### 3.1 Yearly change for the rates of CMA testing in different groups over the 5 years

**FIGURE 1:**
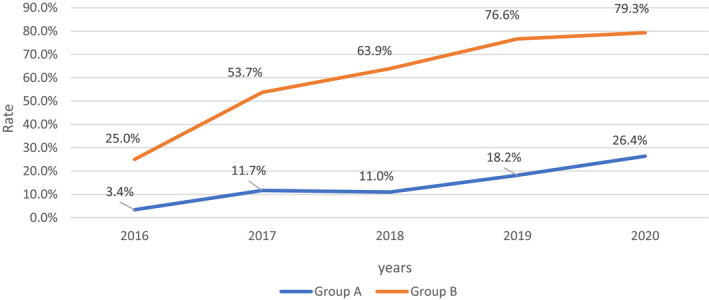
Rate of CMA pregnancies with AMSS solely (group A) and AMSS accompanied by ultrasound anomalies (group B) over 5‐year period. 2016 refers to the period from April to December; 2020 refers to the period from January to May. AMSS, abnormal maternal serum screening; CMA, chromosomal microarray analysis

Of the 3973 cases, 713 (17.9%) were processed in parallel using conventional karyotyping and CMA testing. The rates of CMA testing in group A and group B were 13.2% (476/3600) and 63.5% (237/373), respectively. Regarding the subgroups, the CMA testing values for pregnancies with high risk, critical risk, soft markers and other ultrasound anomalies were 13.0% (461/3540), 25.0% (15/60), 59.5% (156/262) and 73.0% (81/111), respectively. As shown in Figure 1, the yearly rate of CMA testing increased gradually for both group A and group B, from 3.4% in 2016 to 26.4% in 2020 (*P* < 0.05), and from 25.0% in 2016 to 79.3% in 2020, respectively (*P* < 0.05).

### 3.2 Overall CMA results

A total of 85 aberrations were recorded in 82 out of 713 cases processed in parallel using conventional karyotyping and CMA testing. Clinically significant aberrations were observed in 59 (8.3%) cases, involving 59 (69.4%) aberrations. The average number of clinically significant mutations per foetus was 1. VOUS were found in 16 (2.2%) cases, involving 16 (18.8%) aberrations. Only 3 aberrations (Table 2; Case 10, Case 11 and Case 15) were considered novel.

**TABLE 2:** The details of additional CMA findings of clinical significance and uncertain significance in the two groups

Case No.	Additional indications	CMA results	Type of aberration/size	Inheritance	Related syndrome/Pathogenic classification	Outcome
1	/	arr[hg19] 1q21.1q21.2(145,792,037‐147,830,830)x1	del/2.0MB	Deny	Developmental and mental retardation/Likely pathogenic	LB
2	/	arr[hg19] 2q32.3q33.1(195,940,640‐199,623,902)x1	del/3.6MB	De novo	Developmental and mental retardation, characteristic face/Likely pathogenic	TOP
3	/	arr[hg19] 5p15.33p15.32(113,576‐5,216,562)x1	del/5.1MB	De novo	Cri‐du‐chat Syndrome/Pathogenic	TOP
4	/	arr[hg19] 5q23.2(125,979,223‐126,245,903)x3 pat	dup/267KB	Paternal	Adults Leukodystrophy/Likely pathogenic	LB
5	/	arr[hg19] (11)x2 mos hmz	UPD/135MB	/	Russell‐Silver syndrome/Likely pathogenic	TOP
6	/	arr[hg19] Xp22.11p21.2(22520329‐29739422)x1	del/7.2MB	De novo	Autism, mental retardation, developmental retardation, and hypotonia/Likely pathogenic	TOP
7	/	arr[hg19] Xp22.31(6,455,361‐8,135,568)x0	del/1.6MB	Maternal	X‐linked ichthyosis/Pathogenic	LB
8	/	arr[hg19] Xp22.31(6,683,449‐7,887,990)x1	del/1.2MB	Maternal	X‐linked ichthyosis/Likely pathogenic	LB
9	/	arr[hg19] 2p25.3(3,041,821‐4,074,083)x3	dup/1.0MB	Maternal	VOUS	LB
10	/	arr[hg19] 7p15.3(21,584,431‐22,859,461)x3^b^	dup/1.2MB	Paternal	VOUS	LB
11	/	arr[hg19] 7p21.3p21.2(11,227,387‐13,855,151)x3^b^	dup/2.6MB	/	VOUS	LB
12	/	arr[hg19] 15q11.2(22,770,421‐23,276,833)x1	del/506KB	Deny	VOUS	LB
13	/	arr[hg19] 15q11.2(22,770,421‐23,625,785)x1	del/855KB	Deny	VOUS	LB
14	/	arr[hg19] 15q11.2(22,770,421‐23,625,785)x1	del/855KB	Deny	VOUS	LB
15	/	arr[hg19] 16p11.2(32,554,241‐33,863,672)x4^b^	dup/1.3MB	Partly paternal^a^	VOUS	LB
16	/	arr[hg19] 16p12.2(21,740,199‐22,718,351)x1	del/978KB	Deny	VOUS	LB
17	/	arr[hg19] 16q24.2(87,345,448‐87,845,754)x1	del/500KB	Maternal	VOUS	LB
18	/	arr[hg19] 21q21.3(27,301,325‐27,424,959)x3	dup/124KB	De novo	VOUS	Abortion
19	FGR, persistent left superior vena cava, coarctation of aorta	arr[hg19] 2p25.3p11.2(50,813‐87,053,152) hmz,arr[hg19] 2q11.1q37.3(95,550,957‐242,773,583) hmz	ROH/87MB, ROH/147.2MB	Mat UPD	Foetal growth restriction, developmental retardation/Likely pathogenic	LB
20	FGR	arr[hg19] 3q26.33q27.2(182,374,672‐185,041,523)x1	del/2.6MB	De novo	Foetal growth retardation, cardiac anomalies, abnormal digestive system and nervous system; development disorders after birth/Pathogenic	TOP
21	Increased NT, cardiac malformation: VSD, aortic coarctation, increased cardio‐thoracic ratio; FGR; increased spinal curvature, Pulmonary dysplasia	arr[hg19] 5q22.3q23.1(113,627,122‐116,240,273)x1, 8q21.11q21.13(74,350,927‐81,710,386)x1	del/2.6MB, del/7.3MB	De novo	Pathogenic	TOP
22	Wide cavum septum pellucidum, cavum vergae	arr[hg19] 5q35.2q35.3(175,416,095‐177,482,506)x1	del/2.0MB	De novo	Sotos syndrome /Pathogenic	TOP
23	FGR	arr[hg19] 7q11.23(72,650,120‐74,154,209)x1	del/1.5Mb	De novo	Williams‐Beuren syndrome/Pathogenic	TOP
24	Open Spina Bifida, omphalocele, single umbilical artery	arr[hg19] 15q13.2q13.3(31,104,220‐32,444,043)x1	del/1.3MB	Deny	15q13.3 microdeletion syndrome/Likely pathogenic	TOP
25	Small for gestational age; cardiac malformation: Pulmonary stenosis, VSD, aortic straddling	arr[hg19] 16p11.2(29,428,531‐30,190,029)x1	del/623KB	De novo	16p11.2 microdeletion syndrome/Likely pathogenic	TOP
26	Intracardiac echogenic focus	arr[hg19] Xp22.33 or Yp11.32(168,551‐629,999 or 118,551‐579,999)x1	del/461KB	Deny	Asthenospermia and oligospermia/Pathogenic	TOP
27	Small for gestational age	arr[hg19] 10q21.3(68,972,662‐69,925,900)x1	del/953KB	/	VOUS	LB
28	VSD, pericardial effusion	arr[hg19] 13q14.11(43,115,979‐43,733,172)x3	dup/617KB	De novo	VOUS	LB
29	Dandy‐Walker malformation, VSD	arr[hg19] 15q11.2(22,770,421‐23,277,436)x1	del/507KB	Paternal	VOUS	TOP
30	Short humerus	arr[hg19] 15q13.3(32,003,537‐32,444,043)x3	dup/441KB	Deny	VOUS	LB
31	Intracardiac echogenic focus	arr[hg19] 15q11.2(22,770,421‐23,277,436)x1	del/507KB	De novo	VOUS(SL)	LB
32	Tricuspid regurgitation	arr[hg19] 16p13.11(15,510,512‐16,309,046)x3	dup/799KB	Deny	VOUS	LB
33	VSD	arr[hg19] Xp22.31(6,449,836‐8,141,076)x0, (XY)x1	del/1.69MB	Deny	X‐linked ichthyosis/Pathogenic	LB

Abbreviations: CMA, chromosomal microarray analysis; FGR, foetal growth retardation; LB, likely benign; NT, nuchal thickness; SL, susceptibility loci; TOP, termination of pregnancy; VOUS, variants of uncertain significance; VSD, ventricular septal defect.

^a^ Both the parents showed 3 copies of 16q11.2 and normal phenotype.

^b^ Novel aberration.

### 3.3 CMA findings in pregnancies with AMSS only

This group consisted of 3540 foetuses with high‐risk maternal serum screening results and 60 foetuses with critical risk. Amongst the 476 cases analysed using CMA, 43 genetic mutations were observed in 42 cases, and 28 (65.1%) of them detected in 28 cases were clinically significant aberrations. Amongst them, 20 cases were karyotype‐detectable, and 8 were CMA‐detectable only (Table 3). The karyotype‐detectable aberration involved 14 cases of autosomal aneuploidy, 1 case of sex chromosomal aneuploidy, and 5 cases of CNVs sized from 4.3 Mb to 15 Mb. Amongst the 8 cases with CMA‐detectable only results, 1 case of mosaic UPD (11) and 5 cases of CNVs were categorized as likely pathogenic. The remaining 2 cases were considered as pathogenic aberrations, including deletion of 5.1 Mb in the 5p15.33p15.32 region resulting in Cri‐du‐chat syndrome, and deletion of 1.68 Mb at chromosome Xp22.31 resulting in XLI in a male foetus, details of additional CMA findings were presented in Table 2. The incremental yield of clinically significant findings was 1.7% (8/476), and these findings were all observed for high‐risk pregnancies.

**TABLE 3:** The classification and distribution of the abnormal CMA findings in the two groups

	Karyotype‐detectable^a^				Karyotype‐undetectable						
	T13,T18,or T21	SCA	Others	Total	Clinically significant aberrations^a^			VOUS	Incidental findings (including ROH)	Likely benign	Total
					CNVs	ROH	Total				
Group A											
High risk (n = 461)	14 (3.0%)	0 (0.0%)	5 (1.1%)	19 (4.1%)	7 (1.5%)	1 (0.2%)	8 (1.7%)	10 (2.1%)	1 (0.2%)	3 (0.7%)	22 (4.8%)
Critical risk (n = 15)	0 (0.0%)	1 (6.67%)	0 (0.0%)	1 (6.67%)	0 (0.0%)	0 (0.0%)	0 (0%)	0 (0.0%)	0 (0.0%)	0 (0.0%)	0 (0.0%)
Total (n = 476)	14 (2.9%)	1 (0.2%)	5 (1.1%)	20 (4.2%)	7 (1.5%)	1 (0.2%)	8 (1.7%)*,**	10 (2.1%)	1 (0.2%)	3 (0.6%)	22 (4.6%)
Group B											
AMSS + soft markers (n = 156)	9 (5.8%)	2 (1.3%)	1 (0.6%)	12 (7.7%)	2 (1.3%)	0 (0.0%)	2 (1.3%)*	3 (1.9%)	1 (0.6%)	2 (1.3%)	8 (5.1%)
AMSS + other ultrasound anomalies (n = 81)	6 (7.4%)	1 (1.2%)	3 (3.7%)	10 (12.4%)	6 (7.4%)	1 (1.2%)	7 (8.6%)**	3 (3.7%)	0(0.0%)	0(0.0%)	10 (12.4%)
Total (n = 237)	15 (6.3%)	3 (1.3%)	4 (1.7%)	22 (9.3%)	8 (3.4%)	1 (0.4%)	9 (3.8%)	6 (2.5%)	1 (0.4%)	2 (0.8%)	18 (7.6%)

Abbreviations: AMSS, abnormal maternal serum screening; CMA, chromosomal microarray analysis; CNVs, copy number variants; ROH, regions of homozygosity; SCA, sex chromosomal aneuploidy.

^a^ Clinically significant findings.

* *P* > 0.05.

** *P* < 0.05.

Variants of unknown significance were identified in 10 (2.1%) foetuses, amongst which the most frequent aberration was a deletion in the region of 15q11.2, previously categorized as pathogenic/likely pathogenic CNVs.

### 3.4 CMA findings in pregnancies with AMSS and ultrasound abnormalities

A total of 42 genetic mutations were detected in 40 out of 476 cases analysed using CMA and 31 (73.8%) of them detected in 31 cases were aberrations with clinical significance. Of the 31 cases, 22 were detected by karyotyping, including 15 cases of autosomal aneuploidies, 3 cases of mosaic sex chromosomal aneuploidy, 3 cases of imbalanced structural abnormalities and 1 case of triploidy. The incremental yield of CMA regarding clinically significant findings was 3.8% (9/237); the value in pregnancies with ultrasound soft marker was 1.3%, similar to that for pregnancies with solely AMSS (1.3% vs 1.7%, *P* > 0.05). The values for pregnancies with other ultrasound anomalies was 8.6% (7/111), which was significantly higher than that for pregnancies with solely AMSS (8.6% vs 1.7%, *P* < 0.05). Details are presented in Table 3.

The total frequency of VOUS in this group was 2.5% (6/237). The values for pregnancies with ultrasound soft markers and with other ultrasound anomalies were 1.9% and 3.7%, respectively; there were no significant differences between AMSS only and AMSS with ultrasound anomaly groups.

### 3.5 Pregnancy outcomes of foetuses with abnormal CMA findings

Follow‐up information was obtained for 98.8% (81/82) of the foetuses with abnormal CMA findings. All 42 (100%) cases of karyotype‐detectable abnormalities were terminated. The termination rates for pregnancies with karyotype‐undetectable abnormalities of clinical significance and uncertain significance (VOUS) in groups A and B were 50.0% (4/8), 0% (0/10), and 77.8% (7/9), 16.7% (1/6), respectively. The only case of VOUS that ended in termination of pregnancy (TOP) was noted in a foetus with Dandy‐Walker malformation and ventricular septal defect.

## 4 DISCUSSION

In the past years, many studies have emphasized the diagnostic utility of CMA in low‐risk pregnancies.^6^, ^7^, ^12^ The American Congress of Obstetricians and Gynecologists recommends that CMA be made available to all patients choosing to undergo invasive diagnostic testing.^13^ In this study, we evaluated the rate of CMA testing and detection results in pregnancies with AMSS for 5 years. The yearly rates of CMA testing increased over the years. This may be partly associated with patients' increased acceptance of new technology and physicians' improved ability to cope with varying CMA results.

Predictably, trisomy 21 and 18 were the most frequently detected aberrations in pregnancies with AMSS. In addition, 1.7% and 3.8% additional submicroscopic aberrations with clinical significance were found by CMA in group A and group B, respectively. The value of 1.7% was similar to that previously reported for patients with normal ultrasound examinations and normal karyotypes.^5^, ^11^, ^14^, ^15^ It is noteworthy that all the karyotype‐undetectable aberrations in group A were detected from pregnancies with high risk rather than critical risk on maternal serum screening. An abnormal serum screening result frequently indicates a need for NIPT.^16^, ^17^ As demonstrated in this study, if NIPT was offered to pregnant women with abnormal traditional maternal serum screening but without ultrasound anomalies, 13/28 abnormal results with clinical significance would have been missed. However, for concerning pregnancies with critical risk for Down syndrome (1/1000‐1/270), NIPT is more suitable than invasive testing.

Soft markers are generally considered either risk factors for underlying foetal aneuploidy, or only statistical markers for Down syndrome.^18^, ^19^ Therefore, they are actually low‐risk factors for CNVs. In the current study, the proportion of CMA testing for pregnancies with soft markers was much higher than that for women with AMSS only, which indicated that pregnant women with soft markers are more likely to be recommended and willing to undergo CMA testing; however, their incremental yield of clinically significant findings was comparable, although both were much lower than that for women having other foetal abnormalities that had increased chances of being associated with CNVs. Therefore, we believe that the indication of AMSS should receive the same attention as soft markers.

The major challenge of CMA in the prenatal setting is the incremental detection of uncertain results, including VOUS and susceptibility loci (SL), especially in the setting of normal ultrasound. In our laboratory, we generally categorized SL with approximately 10% penetrance as VOUS,^10^ and penetrance of more than 20% as a likely pathogenic variant. Studies exploring choices of women who are undergoing prenatal CMA in a clinical setting regarding these types of findings indicate that most women opt for maximal information on their foetuses and many wish to avoid uncertain results.^20^, ^21^, ^22^ Our policy is to report all findings to the patients to circumvent lawsuits to some extent.^23^ It is worth mentioning that the detection rates of VOUS in AMSS pregnancies with and without ultrasound anomalies showed no significant difference. However, the proportion of clinically significant variants in AMSS with ultrasound anomalies except for soft markers was twice as high as that of VOUS. For the groups with normal ultrasound or with soft markers, the proportions were similar, which was consistent with that found in the study conducted by Stern et al^7^ This further emphasized the importance and value of CMA in the case of ultrasonographic abnormalities, especially structural abnormalities. Nevertheless, most of the additional findings, even those with clinical significance, did not cause TOP after expert genetic counselling. Post‐test counselling in cases with SL is expected to be complicated. In the present study, there were 8 cases harbouring SL, and the most frequently encountered SL was 15q11.2 BP1‐BP2 deletion, the categorization for which was always controversial. The penetrance was previously estimated at 5% to 11%.^9^, ^10^ In a study carried out by Bulter et al,^24^ 15 q11.2 BP1‐BP2 deletion was the most commonly detected genetic aberration in autism patients and was categorized as a likely pathogenic variant in our previous publication.^11^ However, in recent years, several studies supported the viewpoint that this deletion was less likely to have pathogenic effects.^10^, ^25^, ^26^ Some even suggested opting out of such copy number variation reports to both clinicians and couples.^27^ In our study, four foetuses with 15 q11.2 BP1‐BP2 deletion were born and experienced normal development during 1 year of follow‐up after birth, although the infants were still too young to be assessed for neurological development. One case of the deletion was confirmed to be inherited from a parent with a normal phenotype but was finally terminated due to severe ultrasound anomalies. In another case of SL ending in TOP, the aberration was a distal 16p11.2 deletion with 46.8% penetrance^10^ and the ultrasound manifested as heart malformation (Table 2; Case 25). The severity of the ultrasound anomalies plays an important role in the decision‐making for pregnancies receiving variants of uncertain clinical significance results or SL.^28^

The purpose of prenatal diagnosis is not only to make choices about pregnancy, but also to make early predictions of the symptoms that may appear in the foetus after birth and play an essential role in driving early intervention. In our study, we noted aberrations that were considered pathogenic or likely pathogenic but with little clinical significance detected in pregnancies with or without ultrasound anomalies, such as XLI, a recessively inherited disorder of cutaneous keratinization. Male patients always have polygonal, semitransparent and fine scales on the skin at birth or soon after birth, and the scales gradually become deep dark and rough due to a recurrent microdeletion in the stearyl sulfatase gene (STS).^29^ Postnatal follow‐up confirmed the diagnosis of ichthyosis in two male foetuses and revealed normal dermatological manifestations in a female foetus with ichthyosis.^30^

The limitations of this study include its retrospective design with a small sample size; moreover, not all patients with AMSS accepted CMA which may cause some data bias.

In summary, pregnant women with AMSS who underwent invasive prenatal testing only were subjected to the selection process twice via either NIPT or invasive testing, as well as karyotyping only or with CMA currently. The decision to opt for invasive prenatal testing reflected their desires for more definite results for their foetuses. Considering (a) the efficiency to detect additional 1.7% aberrations with clinical significance, (b) the evidence that not all chromosomal anomalies are associated with ultrasound abnormalities, and (c) the continuous accumulation of professional consulting experience for variants of uncertain significance, we conclude that CMA should be offered to all pregnant women with AMSS, especially those with high‐risk results, undergoing prenatal invasive testing, regardless of ultrasound findings.

## CONFLICT OF INTEREST

The authors declare they have no conflict of interest.

## AUTHOR CONTRIBUTIONS

**Xiaoqing Wu:** Data curation (equal); Formal analysis (equal); Funding acquisition (equal); Investigation (equal); Writing‐original draft (equal). **Ying Li:** Data curation (equal); Resources (equal). **Na Lin:** Formal analysis (equal); Resources (equal). **Xiaorui Xie:** Data curation (equal); Resources (equal). **Linjuan Su:** Data curation (equal); Resources (equal). **Meiying Cai:** Data curation (equal); Resources (equal). **Yuan Lin:** Data curation (equal); Resources (equal); Supervision (equal). **Linshuo Wang:** Data curation (equal); Resources (equal). **Meiying Wang:** Conceptualization (equal); Resources (equal). **liangpu xu:** Project administration (equal); Writing‐review & editing (equal). **Hailong Huang:** Methodology (equal); Project administration (equal); Writing‐review & editing (equal).

## Data Availability

The data described in this study is available upon request from the corresponding authors.
